# Exploring stroke survivors' and physiotherapists' views of self-management after stroke: a qualitative study in the UK

**DOI:** 10.1136/bmjopen-2016-011631

**Published:** 2017-03-10

**Authors:** Euan Sadler, Charles D A Wolfe, Fiona Jones, Christopher McKevitt

**Affiliations:** 1 Health Service and Population Research Department, King's Improvement Science, Centre for Implementation Science, Institute of Psychiatry, Psychology and Neuroscience, King's College London, London, UK; 2 Division of Health and Social Care Research, Faculty of Life Sciences and Medicine, King's College London, London, UK; 3 National Institute for Health Research (NIHR) Collaboration for Leadership in Applied Health Research and Care South London, London, UK; 4 National Institute for Health Research (NIHR) Biomedical Research Centre at Guy's and St Thomas’ NHS Foundation Trust and King's College London, Tower Wing Guy's Hospital, London, UK; 5 Faculty of Health, Social Care and Education, Kingston University and St George's, University of London, London, UK

**Keywords:** self-management, QUALITATIVE RESEARCH

## Abstract

**Objectives:**

Stroke is a sudden-onset condition with long-term consequences. Self-management could help address long-term consequences of stroke. Stroke survivors' and health professionals' views of self-management may vary, limiting the successful introduction of self-management strategies. This paper explores stroke survivors' and physiotherapists' views of self-management, focusing on what self-management means, and factors perceived to enable and hinder self-management after stroke, to draw out implications for policy, practice and future research.

**Design:**

Qualitative study using semistructured interviews and a thematic analysis approach.

**Setting:**

Stroke unit and community stroke-rehabilitation services in London, UK.

**Participants:**

13 stroke survivors (8 men and 5 women; aged 53–89 years) admitted to a London stroke unit. 13 physiotherapists: 8 working in an inpatient stroke unit and 5 in community rehabilitation.

**Results:**

Key differences were evident in how self-management was understood between these groups. Stroke survivors were unfamiliar with the term self-management, but most could provide their own definition and relate to the term, and understood it as care of the self: ‘doing things for yourself’ and ‘looking after yourself’. They did not recognise self-management as part of their care, but valued therapists as encouraging experts in supporting their recovery after stroke. Physiotherapists commonly understood self-management as a process in which stroke survivors were expected to take an active role in their rehabilitation and manage their recovery and health, with different understandings of self-management among physiotherapists shaped by the context in which they worked. They reported that individual, social and organisational factors enable and hinder self-management after stroke, with individual and organisational barriers particularly evident in the early stages.

**Conclusions:**

If self-management support approaches are to be used, further work is required to explore the language and strategies used by professionals to support self-management, and the barriers to supporting self-management at different time points after stroke.

**Strengths and limitations of this study**

- A strength of this study is that it explores for the first time the views of self-management after stroke from the perspectives of stroke survivors and physiotherapists.
- The study included stroke survivors from a range of ages and ethnic backgrounds, and physiotherapists with a range of experience in stroke rehabilitation, to ensure that a diversity of perspectives were represented.
- Professional views of self-management after stroke in this study focused on physiotherapists, so future research should include the views of other health and social care professionals working in stroke care.

## Introduction

Stroke is a sudden-onset condition but with long-term consequences, including disability, emotional problems, depression and reduced social participation.^1–4^ Self-management in stroke offers hope of providing ways of addressing long-term consequences. Although self-management is a contested concept with no single definition,^5^ it can broadly be defined as a process in which individuals acquire skills, strategies and knowledge to manage the physical, psychological, emotional and social effects of a chronic condition.^5^ ^6^ A dominant approach underpinning self-management in research and policy^6–10^ is based on the construct of self-efficacy from social cognitive theory.^11^ This emphasises the importance of individuals' belief in their own capability. Self-management programmes based on self-efficacy use strategies to highlight personal resources and support learning. There is evidence that programmes based on self-efficacy principles improve health outcomes.^7–9^ ^12^ However, a number of papers also highlight the inadequacy of this model in that it does not sufficiently consider contextual factors influencing self-management practices.^13^ ^14^ Researchers implementing self-management support approaches have also emphasised the need to embed self-management principles in clinical practice and to address individual, organisational and health system factors within a ‘whole systems approach’,^15^ ^16^ which could address reported difficulties with access for certain groups, for example, minority groups.^17^ However, the evidence of effectiveness of such self-management support interventions on patient and health service outcomes to date remain limited.^18^

Self-management interventions after stroke have been found to reduce disability and depression, and improve self-efficacy, quality of life and social participation based on evaluation of largely short-term measures.^19–23^ However, a greater understanding is required about how professionals construe self-management in the context of different stages of stroke care. It is critical to explore stroke survivor and health professional views if these approaches are to be introduced since there is emerging evidence of differences in perspectives of self-management between health professionals and people with a range of long-term conditions.^24^ ^25^

Physiotherapists work closely with stroke survivors to improve independence in daily activities and quality of life, and stroke survivors regard physiotherapists as playing an important role in their recovery.^26^ ^27^ A limited number of studies focusing on either allied healthcare professional^28^ ^29^ or stroke survivor^30^ ^31^ views of self-management suggest that this process is complex and influenced by individual, social, organisational and cultural factors. If self-management support is to be considered an option for addressing long-term unmet needs, it is important to understand different underlying assumptions between these groups. The aim of this paper is to explore stroke survivors' and physiotherapists' views of self-management after stroke, focusing on what self-management means, and factors perceived to enable and hinder self-management after stroke, to draw out implications for policy, practice and future research.

## Methods

This study used a qualitative interview design to investigate stroke survivors' and physiotherapists' views of self-management after stroke. Participants were recruited via the South London Stroke Register (SLSR), a population stroke register covering an ethnically diverse inner city region.^32^ They were recruited while they were inpatients on a stroke unit at a London hospital in the UK. They took part in longitudinal case studies, as part of a wider study looking at how self-management was understood and is currently introduced and practiced in stroke rehabilitation, focusing on physiotherapy practice as an exemplar case. The wider study involved observations of participants' rehabilitation on the stroke unit and when discharged home, and follow-up interviews with stroke survivors, carers and physiotherapists, who were part of their care. Therefore, stroke survivors were not receiving a specific self-management programme. The first author, a social scientist with experience of conducting qualitative research in healthcare settings and a clinical background in physiotherapy, used purposive sampling to recruit sampling by gender, ethnic background, age and level of function. This was achieved through close liaison with a senior physiotherapist on the stroke unit. Physiotherapists involved in stroke participants' care were recruited by the first author from the inpatient stroke unit and two local community stroke-rehabilitation settings. Interviews took place after stroke survivors were discharged from rehabilitation (ie, 2–6 months poststroke).

All participants were informed in person about the purpose of the study, and that taking part would be voluntary and information kept confidential. Stroke survivors who were medically unstable, had severe communication or cognitive impairments, or were unable to understand English were not included in the study. All participants provided their informed written consent prior to taking part.

Stroke participants recruited to the wider study (N=17) were observed participating in rehabilitation, and subsequently invited to take part in semistructured interviews conducted by the first author. Of these 17 participants, 13 consented to take part (one participant declined; one died; one was lost to follow-up and one experienced a decline in cognitive impairment). Stroke survivors were all interviewed in their own home. In seven interviews, carers were also present. Physiotherapists were interviewed in their workplace, either in a quiet room on the stroke unit or in a community rehabilitation setting. All interviews were audio-recorded with participant consent. A topic guide of questions was used during the interviews. With the exception of exploring the impact of the stroke with stroke participants only, similar questions were asked about stroke survivors' and physiotherapists' views of self-management after stroke. These included how they understood self-management, perceptions of self-management support provided by physiotherapists and factors perceived to enable and hinder self-management after stroke (see box 1). To aid understanding, we provided stroke participants with a broad definition of self-management as ‘learning to do things for yourself’. This strategy was used to address the likelihood of them being unfamiliar with the term, which has been reported elsewhere.^30^ ^31^ Participants were not educated on the concept of self-management before interviews were conducted. Field notes were taken during interviews to aid later interpretation of the interview transcripts. Data saturation was reached through an iterative process of data collection and analysis in which the first author perceived that no new themes were emerging.

Box 1 **Topic questions used in the interviews**

Stroke survivors

Have you heard of the term self-management? What do you think it means? (define broadly as ‘learning to do things for yourself’)

What are your views on self-management after a stroke?

In what ways did physiotherapists help you to learn to do things for yourself in hospital? What about when you came home?

What has helped you to learn to do things for yourself since the stroke?

What has not helped you to learn to do things for yourself since the stroke?

Physiotherapists

How would you define the term self-management?

What are your views on self-management after stroke?

What self-management support would you as a physiotherapist be involved in providing to stroke survivors?

What factors might help stroke survivors and their carers to learn self-management activities after stroke?

What factors might hinder stroke survivors and their carers to learn self-management activities after stroke?

Interview data were transcribed, imported and sorted in NVivo (V.X7). A thematic analysis of interviews^33^ was then conducted by the first author. This involved close reading of interview transcripts and coding for themes. Physiotherapist and stroke survivor interview transcripts were first coded separately. A coding tree was constructed of themes emerging from the interview data. The ‘one sheet of paper method’^34^ was then used to visually map out and synthesise themes and relationships between themes, and compare similarities and differences between the two groups. This was followed by discussion and interpretation of the coding tree by two authors (ES, CM), and subsequent refinement of themes, to ensure quality and rigour of the analysis process.^35^

## Results

### Sample

The sample comprised 26 participants: 13 stroke survivors and 13 physiotherapists (tables 1 and 2, respectively). Stroke participants were aged between 53 and 89 years (mean age 71 years), 2–6 months poststroke; eight were men and five were women. Although nearly equal numbers of men and women were recruited to the wider study, more women declined to be interviewed. Of stroke participants, seven were White British/Irish, four Black Caribbean and two Black African. Most reported a good level of physical recovery following their stroke; nine walked with a stick or crutch, while three used a frame, and one person used a wheelchair. The majority had other impairments following the stroke, including reduced communication, mood and ongoing fatigue. Physiotherapists had a range of experience in stroke rehabilitation (between 1 and 7+ years); eight worked on the inpatient stroke unit and five in community stroke-rehabilitation (table 2). Interviews lasted between 30 and 90 mins.

**Table 1:** Stroke participant characteristics

Participant	Age	Gender
1	54	Male
2	77	Male
3	75	Male
4	53	Male
5	76	Male
6	66	Female
7	59	Male
8	56	Male
9	89	Female
10	63	Male
11	80	Female
12	82	Female
13	89	Female

**Table 2:** Physiotherapist characteristics

Participant	Location worked	Experience in stroke rehabilitation (years)
Physiotherapist 1	Inpatient stroke unit	2
Physiotherapist 2	Inpatient stroke unit	1
Physiotherapist 3	Community rehabilitation	2
Physiotherapist 4	Community rehabilitation	2
Physiotherapist 5	Inpatient stroke unit	3
Physiotherapist 6	Inpatient stroke unit	3
Physiotherapist 7	Inpatient stroke unit	3
Physiotherapist 8	Inpatient stroke unit	3
Physiotherapist 9	Community rehabilitation	5
Physiotherapist 10	Community rehabilitation	7+
Physiotherapist 11	Community rehabilitation	7+
Physiotherapist 12	Inpatient stroke unit	7+
Physiotherapist 13	Inpatient stroke unit	7+

### Stroke survivors' and physiotherapists' views of self-management

Themes, and related subthemes, pertaining to stroke survivors' and physiotherapists' views of self-management were: (1) meanings of self-management: ‘doing things for yourself’ and ‘looking after yourself’; taking an active role in rehabilitation and managing one's recovery and health; and (2) factors enabling and hindering self-management and early recovery after stroke: quality and nature of therapist/patient relationship and communication; support from family members; and individual and organisational factors. In reporting themes, free-text response categories are sometimes discussed using frequencies. In supporting quotations, ‘…’ indicates an omission of text not relevant to content; abbreviations I and P stand for ‘interviewer’ and ‘participant’, respectively; and authorship of quotations are provided at the end, with work location for physiotherapists and gender and age for stroke survivors.

### Meanings of self-management

#### ‘Doing things for yourself’ and ‘looking after yourself’

As has been reported elsewhere,^30^ ^31^ stroke survivors were unfamiliar with the term self-management, but most could provide their own definition and relate to the term. Nine out of 13 participants, including men and women and those from different ethnic backgrounds, understood self-management as a process of ‘doing things for yourself’. This reflected an ability to resume everyday activities prior to the stroke, such as dressing, bathing and cooking, but also participating more broadly in social roles, including managing one's household and finances, and returning to work, without relying on help from others:

Self-management is like cooking your own food, doing everything for yourself…washing your own face, putting on your own clothes, buttoning up your shirt and taking off your shoes, tying your shoe laces, things like that, without anybody helping you. (Stroke participant 10, male, 63 years)

P: That [self-management] means managing your own money. …That I can continue paying my bills, my rent, anything I have to pay and I can buy what I want.

I Anything else?

P Managing my household.

I What do you mean?

P Know what I want to bring into the home, like food shopping and things like that. (Stroke participant 6, female, 66 years)

Self-management in relation to ‘doing things for yourself’ was also understood by three White British men, with varying levels of physical function following their stroke, as making healthy lifestyle changes to enhance their future recovery. Such changes included eating well, getting sufficient sleep and avoiding risks when walking outside. Two of these men further perceived self-management as taking responsibility for health maintenance, including attending to one's self-appearance, dressing well and maintaining personal hygiene, as one man said:

P Well it's [self-management] about appearance and hygiene.

I Why would you say that?

P Well I think it's appearances that count. I wouldn't like to go out…if I wasn't washed properly. And putting on clean clothes and things like that. It all comes into it. (Stroke participant 7, male, 59 years)

In addition, self-management was interpreted by two men and women, from different ethnic groups, as part of a process of ‘looking after yourself’, reflecting a person's attitude to overcome the impact and consequences of an adversity such as stroke. This involved positive attitudes, such as self-determination, ‘inner strength’ or adopting a stoical attitude to ‘get on with life’, as one woman said:

Self-management is about looking after yourself. … It's no good sitting feeling sorry for yourself all the time is it? That's what it boils down to with me I think. It doesn't get you anywhere does it to sit and mope. (Stroke participant 11, female, 80 years)

#### Taking an active role in rehabilitation and managing one's recovery and health

In contrast, physiotherapists commonly viewed self-management as a process in which stroke survivors were expected to take an active role in their rehabilitation and manage their recovery and health. Typically, physiotherapists described providing self-management support as teaching stroke survivors technical skills, including strategies to monitor body postures, tailored exercise programmes, practicing functional tasks and walking regimes and the correct use of splints to support weaker limbs. Evident in physiotherapists' accounts were implicit notions of personal responsibility and compliance with professional advice.

Self-management, I suppose it's, in terms of a patient, taking an active role in their own rehabilitation, not just in terms of participating in our sessions, but also outside our sessions, taking on board what advice we're giving. And being able to continue with that and doing those sort of exercises, programme, taking a bit more of a role in their recovery and their rehabilitation. (Physiotherapist 5, stroke unit)

In line with a dominant self-efficacy model of self-management,^6^ physiotherapists often articulated the positive psychological characteristics perceived to be necessary to take an active role in their rehabilitation and recovery after stroke. These included an ability to acquire knowledge about the stroke, problem solve, set goals, recognise needs, manage changes over time and seek appropriate professional support. For two physiotherapists working on the stroke unit, adopting such a proactive approach to managing needs extended beyond the stroke, to managing any health condition, as one therapist said:

Self-management is being able to manage yourself and manage any condition that you suffer with…being able to recognise what needs you have, being able to do something proactive about those needs, so contacting the services that you need. (Physiotherapist 12, stroke unit)

Self-management was understood by two community physiotherapists as incorporating not only an ability to manage the immediate physical effects of the stroke but also longer term consequences. This included medication regimes, daily routines and activities, and the psychological and emotional effects of the stroke.

Self-management it's not just talking about more physio, but more holistically. They've got to manage post stroke, how they are feeling in relation to activities, but also their mood, their future, what medication they may be on as well. So it's kind of the bigger picture. (Physiotherapist 4, community rehabilitation)

In summary, key differences were evident in how self-management was understood between these two groups. Stroke survivors were unfamiliar with the concept of self-management, but viewed it as care of the self: ‘doing things for yourself’ and ‘looking after yourself’. Physiotherapists commonly saw self-management as a process in which stroke survivors were expected to take an active role in their rehabilitation and manage their recovery and health. The results suggest that professionals need to be able to adapt and find a common ground with stroke survivors and their underlying assumptions about self-management.

### Factors enabling and hindering self-management and early recovery after stroke

Stroke survivors identified factors related to care and support perceived to enable their early recovery after stroke, rather than self-management per se, particularly in terms of the quality of interpersonal relationships with therapists and family support, whereas physiotherapists spoke about a number of factors enabling and hindering self-management after stroke.

#### Quality and nature of therapist/patient relationship and communication

Over half of stroke participants, including men and women of different ethnic backgrounds, emphasised the quality and nature of relationship and communication, often with a named physiotherapist, as a key component of their care enabling their early recovery. They valued therapists, on the stroke unit and when discharged home, as encouraging experts with particular knowledge and expertise, who had been respectful and empathetic during their rehabilitation, seeing this as an expected part of their care rather than self-management specifically. For example, one man said:

The way she talks…to make sure I do what I am supposed to do, and being so friendly as well. She's exceptional. (Stroke participant 5, male, 76 years)

In contrast, the nature of the therapist/patient relationship was considered by physiotherapists working in community settings, but not among those on the stroke unit, as a prerequisite for the success of supporting self-management practices after stroke. They spoke about their role as being more akin to a ‘guide’, core to which was the development of collaborative relationships with stroke survivors, as part of a person-centred care approach to facilitate self-management. This was through fostering shared decision-making and the provision of tailored information and education, for example, in terms of goal setting to improve further recovery:

Well I suppose we're seen as the experts, but I feel that we're there to guide and support people rather than dictate. So I think that's where education comes into play why we sort of say this is where you are now, where would you like to get to? That's the bit that's patient led, and how do you feel that you're going to get there? (Physiotherapist 9, community rehabilitation)

#### Support from family members

Whereas a self-management ethos aims to promote confidence, knowledge and skills among people with long-term conditions,^36^ when stroke survivors talked about family support following discharge from hospital, over half described a dependence on family members for practical and emotional support following their stroke. This included support with washing, dressing, cooking and shopping. Accounts suggested that such support might have been a barrier to learning self-management practices in the context of striving for independence in everyday activities in the early stages after stroke, as one man said:

I: How has your wife helped you?

P: Well she packed up work to take care of me.

I: So is she here all the time?

P: All the time, so the only time she's not here is when she goes out shopping or something, like I've got family come round when she goes out. (Stroke participant 1, male, 54 years)

All physiotherapists, however, felt that self-management was influenced by social support provided by family carers, and recognised that the quality and availability of such support varied, potentially enabling or hindering self-management practices. Whereas for stroke survivors, support meant family members doing things for them, therapists perceived they were there to reinforce the message of self-reliance. Recruitment of family carers appeared to be a strategy among physiotherapists to encourage stroke participants' adherence to professionally taught exercise programmes, rather than providing tailored support to enable family members in their caring role.

You can maximise somebody's rehab if you know that they've got friends or family that will do appropriate things with them out of designated therapy time but then they're the ones that will be long-term with that person if they have ongoing management needs. (Physiotherapist 2, stroke unit)

So you're trying to encourage self-management, part of it was actually trying to encourage team work with her [stroke survivor's] son, and guidance to her and her son on how they can do these things while we're away and when we leave. (Physiotherapist 3, community rehabilitation)

#### Individual and organisational factors

Physiotherapists, but not stroke survivors, also reported other individual and organisational factors hindering self-management after stroke. Commonly, individual factors were psychological problems and cognitive deficits among stroke survivors, including a lack of motivation, confidence, an ability to take responsibility as well as stroke-related fatigue and cognitive impairment. This led some physiotherapists to question the readiness and capacity of stroke survivors to learn to self-manage, which has been reported elsewhere,^29^ and in this study a perceived dependence on professional support during rehabilitation. For example, one community physiotherapist said:

In the beginning people are just really low in confidence, they're like shell shocked, they don't know what to do and when we set goals with patients, they often say ‘oh well, you're the professional, you tell me what to do’, so they're not ready to self-manage or to take responsibility themselves yet…they very much look at the healthcare professional to guide them. (Physiotherapist 11, community rehabilitation)

Most physiotherapists considered that self-management support was challenging to apply on the inpatient stroke unit since expert-led medical treatment and rehabilitation tended to reinforce the sick role, and the hospital was under pressure to discharge patients quickly. This differed from community settings which were perceived by therapists to be more conducive to supporting self-management practices in stroke survivors' own home environment. However, physiotherapists did not articulate how to try to overcome the barriers presented by an acute medical environment.

When they're in the hospital and the hospital is looking after them, the hospital is dealing with all the problems, they don't necessarily have to think about that [self-management] as much as when they are actually having to put on a pad, to put on a splint which can be difficult for some people. (Physiotherapist 7, stroke unit)

In summary, a range of factors were perceived by physiotherapists to enable and hinder self-management after stroke, with individual and organisational barriers, particularly evident in the early stages. Stroke survivors did not recognise self-management as part of their care, but particularly valued therapists as encouraging experts in supporting their early recovery after stroke. Unlike therapists, this is likely to be related to stroke survivors' difficulties conceptualising self-management as a specific component of their rehabilitation and care at this early stage.

## Discussion

This UK study is the first study to explore stroke survivors' and physiotherapists' views of self-management after stroke. We found key differences in how self-management was understood and perceived in practice between these groups. As reported elsewhere,^30^ ^31^ stroke survivors were unfamiliar with the term self-management, but nevertheless, most could provide their own definition and relate to the term. Meanings of self-management pointed to ideas related to care of the self in terms of ‘doing things for yourself’ and ‘looking after yourself’. They did not recognise self-management as part of their care, but valued therapists as encouraging experts, expecting the expert to look after them and support them in their recovery following the stroke. In contrast, physiotherapists commonly viewed self-management as a process in which stroke survivors were expected to take an active role in their rehabilitation and manage their recovery and health. This resembled features of a dominant approach to self-management based on self-efficacy principles in the health research and policy literature.^6–10^

A number of individual, organisational and social factors were perceived by physiotherapists to enable and hinder self-management after stroke, concurring with other studies focusing on allied healthcare professionals' perspectives.^28^ ^29^ In our study, some physiotherapists questioned stroke survivors' ability to self-manage and take responsibility for their recovery early after stroke, which shaped a perceived dependence on health professionals. The reasons for this might include stroke-related cognitive impairments and fatigue, which has been similarly reported in a Dutch study.^29^ We also found that the biomedical context and organisational practices of the acute medical environment of the stroke unit were perceived by therapists to be barriers to supporting self-management at this early stage.

Our study has shown how different understandings of self-management among physiotherapists were shaped by the context in which they worked. Therapists on the stroke unit commonly understood self-management within an individualistic framework, whereas those in community settings constructed broader notions of self-management based on collaborative partnership working. The latter has similarly been reported in other studies among health professionals working in community settings with people with a range of long-term conditions.^9^ ^37–39^ In our study, one explanation for this is that outside the biomedical context of the stroke unit setting, health professionals may be more likely to endorse a social model of self-management incorporating an ethos of person-centred care that is more ‘responsive to individual patient preferences, needs, and values’ (ref. 40, p. 40).

A recent review supports the finding that stroke survivors report the quality of communication with health professionals as a core component of self-management support.^41^ We found that stroke survivors perceived the quality of interpersonal relationships as key to good quality of care rather than self-management specifically, placing value on therapists as encouraging experts with particular expertise. This has been found to be a prerequisite for the success of self-management of long-term conditions in the literature.^9^ ^39^ Studies among people with a range of long-term conditions, including diabetes,^42^ ^43^ multiple sclerosis,^44^ cancer^45^ and multimorbidity,^46^ report the importance of developing collaborative partnerships with health professionals to enable self-management practices. However, this was not explicitly expressed among stroke survivors in our study. One possible explanation is that although most stroke survivors reported a good level of recovery, because of the sudden onset and disabling nature of stroke,^47^ they were not ready to think about self-management but depended on health professionals as experts to support them, which may differ from other progressive or variable long-term conditions.

### Strengths and weaknesses of the study

We acknowledge there were some limitations to our study, including the small sample size, that stroke participants were mostly a well-recovered cohort, and focusing on only one group of health professionals. In interviewing stroke survivors 2–6 months poststroke, it is also likely that responses were influenced by reduced recall among some participants. However, the strengths of the study were that we included stroke survivors with a range of ages, from different ethnic backgrounds, and physiotherapists with a range of experience in stroke rehabilitation, ensuring a diversity of perspectives were represented.

## Conclusions and implications for policy, practice and future research

This study has a number of implications for policy, practice and future research. First, we have identified differences in how self-management is understood and perceived in practice between these two groups that may limit its implementation and adoption. It is difficult for health professionals to support self-management in practice when stroke survivors have different views of the term and its value in their early recovery after stroke. Therapists need to explore any such differences with stroke survivors, explain what self-management is and its value to their care, rehabilitation and early recovery after stroke before encouraging them to self-manage. Our findings could benefit from further studies to ascertain their replication. Second, our study focused on physiotherapists as an exemplar case. Given the multidisciplinary team nature of stroke rehabilitation, future research should include the views of a range of health and social care professionals working in stroke care. Finally, if self-management support approaches are to be used, further work is required to explore the language and strategies used by professionals to support self-management, and the barriers to supporting self-management at different time points after stroke.
